# Evaluating the immediate and delayed effects of psychological need thwarting of online teaching on Chinese primary and middle school teachers’ psychological well-being

**DOI:** 10.3389/fpsyg.2022.943449

**Published:** 2022-08-16

**Authors:** I-Hua Chen, Xiu-mei Chen, Xiao-ling Liao, Ke-Yun Zhao, Zhi-Hui Wei, Chung-Ying Lin, Jeffrey Hugh Gamble

**Affiliations:** ^1^Chinese Academy of Education Big Data, Qufu Normal University, Qufu, China; ^2^Faculty of Education, Qufu Normal University, Qufu, China; ^3^International College, Krirk University, Bangkok, Thailand; ^4^School of Communication, Qufu Normal University, Rizhao, China; ^5^Department of Development and Research, Shanghai Open University, Shanghai, China; ^6^Institute of Allied Health Sciences, College of Medicine, National Cheng Kung University, Tainan, Taiwan; ^7^Department of Occupational Therapy, College of Medicine, National Cheng Kung University, Tainan, Taiwan; ^8^Biostatistics Consulting Center, National Cheng Kung University Hospital, College of Medicine, National Cheng Kung University, Tainan, Taiwan; ^9^Department of English, National Changhua University of Education, Changhua, Taiwan

**Keywords:** psychological need thwarting, online teaching, longitudinal data, instrument validation, COVID-19 pandemic, teacher psychology, psychological well-being, burnout

## Abstract

Recent studies on the effects of mandatory online teaching, resulting from the COVID-19 pandemic, have widely reported low levels of satisfaction, unwillingness to continue online teaching, and negative impacts on the psychological well-being of teachers. Emerging research has highlighted the potential role of psychological need thwarting (PNT), in terms of autonomy, competence, and relatedness thwarting, resulting from online teaching. The aim of this study was to evaluate the immediate and delayed (longitudinal) effects of PNT of online teaching on teachers’ well-being (including distress and burnout), intention to continue online teaching, and job satisfaction. Moreover, data collected from both cross-sectional and longitudinal surveys allowed for a systematic validation of an important instrument in the field of teacher psychology, the Psychological Need Thwarting Scale of Online Teaching (PNTSOT), in terms of longitudinal reliability and validity. The data reveal the usefulness of the construct of PNT in terms predicting and explaining teachers’ willingness to continue using online teaching as well as the degree of burnout after a period of 2 months, such that PNT is positively associated with burnout and negatively associated with willingness to continue online teaching. As such, the PNTSOT is recommended for future research evaluating the long-term psychological, affective, and intentional outcomes stemming from teachers’ PNT. Moreover, based on our findings that the impact from PNT of online teaching is persistent and long-term, we suggest that school leaders provide flexible and sustained professional development, model respectful and adaptive leadership, and create opportunities for mastery for the development of community of practice that can mitigate the thwarting of teachers’ autonomy, competence, and relatedness during times of uncertainty. Additionally, in terms of the psychometric properties of the PNTSOT instrument, our empirical findings demonstrate internal reliability, test–retest reliability, measurement invariance, and criterion validity (concurrent and predictive) based on cross-sectional and longitudinal data.

## Introduction

The COVID-19 pandemic has had a profound impact on the world, with pervasive effects in all aspects of life, including education. In fact, according to a survey by the United Nations Educational, Scientific and Cultural Organization (UNESCO), more than 180 countries had closed all school campuses during the pandemic, affecting the lives of 1.6 billion primary and secondary school students (UNESCO, 2020). This large-scale impact on a significant portion of the population has made the ongoing and lasting impacts of the COVID-19 pandemic of increasing concern to educational practitioners and researchers in terms of both short-term and long-term outcomes (Chen et al., 2021; Alhazmi et al., 2022; Haug et al., 2022). In response to the urgent demands and concerns of educators, several international organizations published special reports to provide the guidelines or frameworks for policy-makers, educators, and other stakeholders [e.g., The World Bank (Rodriguez et al., 2021), Organisation for Economic Co-operation and Development (Gouëdard et al., 2020), and UNESCO (2020)]. One common theme in these reports is an emphasis on the effective use of technology as a medium for teachers to shift instruction from physical to online learning environments as a primary and immediate adaptation to the crisis (Munoz-Najar et al., 2021; Statistics for National Statistics, 2021). Over time, researchers and practitioners began to share successful online teaching models (or experiences) adopted by schools in various countries during the closure of school campuses, with optimism that the pandemic may serve as an opportunity for reimagining the future of education by promoting teachers’ innovative instruction and enhancing teachers’ ability to effectively and appropriately integrate technology into teaching (Mishra et al., 2020; Shamir-Inbal and Blau, 2021).

### Unexpected side effects of mandatory online teaching

The sudden onset of the pandemic required teachers to adopt online teaching, generally with very little training or background experience in distance learning (Silva et al., 2021; Trust and Whalen, 2021; Yi et al., 2021). Conceptually, the promotion of teachers’ digital competencies brought about by the required use of both synchronous (SCMC) and asynchronous (ACMC) computer mediated communication held the potential to bring about the positive outcomes for teaching and learning associated with technology-integrated instruction (Bank, 2021; Hilger et al., 2021; Rodriguez et al., 2021). However, in reality, the outcomes of online teaching were perceived by teachers and other stakeholders to be largely negative, with empirical studies revealing that the hasty implementation of online courses by teachers with insufficient training or experience led to perceived high barriers related to the use of technology for teaching (Trust and Whalen, 2021) and resulting low teaching satisfaction (Fauzi and Khusuma, 2020), high workload (including work stress; Aperribai et al., 2020; Richmond et al., 2020; Jelińska and Paradowski, 2021a,b), and reduced willingness to use online teaching in the future (Zheng and Song, 2020). The title of a recent publication, “E-learning? Never again!” (Kulikowski et al., 2021), aptly describes the situation wherein instructors in higher education were required to implement online courses with unintended consequences of decreased job satisfaction, work motivation, and job involvement. In explaining how the online teaching required by COVID-19 impacted work motivation, core job characteristics were evaluated by Kulikowski et al. (2021); among these, four characteristics (task identity, task significance, task autonomy, and social interaction) decreased during online teaching, thereby leading to the unintended negative impacts on teachers’ work motivation. Other empirical studies have revealed that mandatory online teaching contributed to detrimental side effects, particularly in terms of autonomy, due to work overload and inadequate working environments (Chan et al., 2021; Hilger et al., 2021; Soncini et al., 2021; Vargas Rubilar and Oros, 2021). More specifically, a significant decrease in work-related autonomy was found among Germany schoolteachers after the onset of pandemic (Hilger et al., 2021). Likewise, Italian teachers reported that, during mandatory online teaching, an increased workload and difficulties in carrying out teaching activities were main threats related to practical aspects of teaching (Soncini et al., 2021), while Argentine teachers reported that the work schedule for mandatory online teaching was disorganized (Vargas Rubilar and Oros, 2021), reflecting the lack of task autonomy (Kulikowski et al., 2021). In addition to threats to task autonomy, qualitative data from American elementary schoolteachers revealed that more freedom in the performance of tasks was required during mandatory online teaching and that the result of the standardization of distance learning was characterized by “having their hands tied behind their backs” (Chan et al., 2021).

While the findings by Kulikowski et al. (2021) were from teachers in higher education, it is reasonable to infer that primary and middle school teachers may have encountered similar impacts to work motivation, since teachers at the primary and secondary level also had very little online teaching experience before the outbreak of the pandemic (Silva et al., 2021; Yi et al., 2021). Moreover, studies have shown that primary and middle school teachers may be even more reluctant to conduct online teaching or refuse to engage in online teaching while quarantine measures are in effect, as compared to teachers in higher education contexts (Jelińska and Paradowski, 2021a,b). As such, we contend that primary and middle school teachers represent a vulnerable population that deserves greater attention and research in terms of the psychological effects of online teaching during the pandemic, due to the several adverse effects reported in the literature (Chan et al., 2021; Ozamiz-Etxebarria et al., 2021b; Silva et al., 2021; Soncini et al., 2021; Vargas Rubilar and Oros, 2021).

### Psychological need thwarting of online teaching

The emerging construct of psychological need thwarting (PNT) has been used to describe the effects of online teaching on primary and middle school teachers (Chen et al., 2020; Yi et al., 2021), finding significant effects from PNT of online teaching on psychological well-being. The construct of PNT was developed on the basis of Self Determination Theory (SDT), which includes three basic psychological needs – autonomy, competence, and relatedness – which strongly influence an individual’s well-being (Ryan and Deci, 2000; Deci and Ryan, 2015). As such PNT serves as a more appropriate construct, as compared to Hackman and Oldham’s Job Characteristics Theory (JCT), as applied in the study by Kulikowski et al. (2021). While JCT focuses on work motivation, the scale is largely descriptive in nature. In comparison, the variables of PNT have greater potential to interpret and elaborate the mechanisms behind the impact of online teaching on teachers’ mental health. A better understanding of these underlying mechanisms can provide a more practical and nuanced contribution by evaluating the relationships among critical factors associated with teachers’ psychological well-being during COVID-19 – an area of increasing importance to scholars in the field (Sahu, 2020; Palma-Vasquez et al., 2021; Yi et al., 2021; Jelińska and Paradowski, 2021a,b).

To extend our understanding of the dynamics of teacher psychology during online teaching, a more complete analysis of the long-term effects of online teaching on psychological well-being is required, beyond the relatively superficial findings concerning teachers’ frustration (e.g., technological barriers, lack of willingness to engage in online teaching, and dissatisfaction) already covered sufficiently in the literature (Aperribai et al., 2020; Fauzi and Khusuma, 2020; Richmond et al., 2020; Zheng and Song, 2020; Trust and Whalen, 2021; Jelińska and Paradowski, 2021a,b). As such, while some preliminary investigations have demonstrated that schoolteachers were substantially impacted by mandatory online teaching in terms of psychological distress, the underlying mechanisms contributing to teacher psychology are still largely unknown. For example, while studies have shown that more than half (58.27%) of primary and secondary school teachers reported poor mental health during school closures (32) or that 20-26% of teachers reported mental health issues related to anxiety and depression occurred during quarantine (Yi et al., 2021), specific risk or protective factors related to teachers psychological well-being have yet to be clearly delineated in the extant literature.

The potential of PNT in explaining the mechanisms behind the impact of online teaching on teacher psychological well-being has some support from recent research (Yi et al., 2021). While most studies have focused on the satisfaction of individuals’ psychological needs, research into environments which fail to satisfy (e.g., frustrate or block) these psychological needs has resulted in a complementary, but conceptually distinct, phenomenon to need satisfaction; namely, psychological need thwarting or PNT (Bartholomew et al., 2011; Gunnell et al., 2013; Cuevas et al., 2015). The literature has demonstrated that psychological need satisfaction and PNT involve different psychological processes, and importantly, that PNT exerts a stronger influence on an individual’s negative affect as compared to psychological need satisfaction (Gunnell et al., 2013; Huyghebaert et al., 2018; Ebersold et al., 2019). As such, given the environment created through mandatory online teaching during the pandemic, it is reasonable to surmise that the perspective of PNT resulting from online teaching can better provide a more comprehensive explanation and elaboration of the influences of online teaching on teachers’ motivation, willingness to conduct online teaching, and psychological well-being.

While the value of PNT in terms of psychological well-being has been reported in the literature, to date there have been few studies conducted during school closure which have examined the association between PNT related to online teaching (or subscales of PNT, such as autonomy-thwarting) and psychological well-being among primary and middle schoolteachers (Collie, 2021; Yi et al., 2021). One study adopted SDT as a framework to examine the effect of intrinsic motivation, extrinsic motivation, and TPK (technological and pedagogical knowledge) self-efficacy on the intention to continue using online teaching, mediated by the variable of burnout, but did not directly evaluate specific PNT factors (Panisoara et al., 2020). Thus, while these studies have provided preliminary support for the potential negative effects of PNT related to online teaching, data were collected only during school closure periods. This leads to an interesting, and heretofore unanswered, question: what are the lasting, long-term influences of PNT of online teaching on both school teachers’ future intention to conduct online teaching and psychological well-being, particularly after online teaching is no longer required? The answer to this question is of vital importance, as the thwarting of teachers’ psychological needs may greatly reduce both immediate and long-term intention of using online teaching, potentially impacting teachers’ overall attitudes towards the use of technology for teaching. Therefore, for a thorough evaluation of the impact of PNT from online teaching on both immediate and long-term intention and psychological well-being, data from different time points adopting a longitudinal design are necessary.

### The measure of teachers’ psychological need thwarting

In the specific context of the COVID-19 pandemic, PNT, particularly related to online teaching, has been developed and validated to some extent (Yi et al., 2021). However, compared to the construct of psychological need satisfaction, which has received greater attention, PNT is still an emerging construct (Huyghebaert et al., 2018; Wu et al., 2018; Lagios et al., 2022). As such, validation of instruments for the measurement of PNT is a prerequisite for conducting further studies in this area. According to a review of the literature, Bartholomew et al. (Bartholomew et al., 2011) first developed a scale to measure individual PNT in the context of sports (i.e., Psychological Need Thwarting Scale, PNTS) and later adapted this scale for the evaluation of a teaching environment; namely, a teachers’ version of the Psychological Need Thwarting Scale (TPNTS; Bartholomew et al., 2014). The TPNTS has been translated into different languages, including a Spanish version (Cuevas et al., 2015) and a Chinese version (Chen et al., 2020). Recently, during the COVID-19 outbreak, in order to measure teachers’ PNT related to the implementation of online teaching, Yi et al. (Yi et al., 2021) made necessary revisions to the Chinese version of the TPNTS to address the context of mandatory online teaching [i.e., Psychological Need Thwarting Scale of Online Teaching (PNTSOT)].

The TPNTS and PNTSOT instruments (Bartholomew et al., 2014; Cuevas et al., 2015; Chen et al., 2020; Yi et al., 2021) are based on Bartholomew et al.’s (2011) framework for PNT, and include 12 items equally divided into the three dimensions of psychological need thwarting: autonomy, competence, and relatedness. Regarding to the quality of the instruments, TPNTS and PNTSOT have suitable internal reliability with Cronbach’s alphas ranging from 0.76 to 0.90. Moreover, factorial validity has been established by several studies through the use of confirmatory factor analysis (CFA; Cuevas et al., 2015; Chen et al., 2020; Yi et al., 2021). In support of the instruments’ criterion validity, significant positive relationships of PNT with teacher burnout (Bartholomew et al., 2014; Cuevas et al., 2015; Chen et al., 2020; Franco et al., 2022) and psychological distress (Yi et al., 2021) have been found. Despite the above evidence, showing satisfactory reliability and validity for these scales, predictive validity testing has yet to be conducted in terms of the association between PNT of online teaching and both the intention to continue using online teaching in the future and the delayed effects on longer-term psychological well-being.

### Aims of the present study

Online teaching has shown the potential to decrease task identity, task significance, autonomy, and social dimensions of teachers’ job characteristics (Kulikowski et al., 2021). We believe this finding, of a negative impact of online teaching on teachers’ job characteristics, may be best explained by an environment that thwarts individuals’ psychological needs. Likewise, the thwarting of teachers’ psychological needs arising from mandatory online teaching may also explain the moderate level of psychological distress (Palma-Vasquez et al., 2021; Yi et al., 2021) and very low satisfaction or intention to engage in online teaching (Fauzi and Khusuma, 2020; Zheng and Song, 2020) among school teachers reported in other COVID-19 studies. Considering the above points, we believe that it is essential to evaluate the PNT of online teaching and treat PNT as a warning (risk factor) for predicting potential burnout, considering the empirically demonstrated role of PNT as a mediator between a stressful environment and teacher burnout (Franco et al., 2022). As such, PNT can serve as a predictor of burnout, providing valuable insights for the design of interventions to address teachers’ psychological needs before burnout occurs, preventing the associated negative effects on intention to continue online teaching and long-term psychological well-being. The contribution of this predictive role of PNT of online teaching can assist in overcoming the current difficulties in effectively evaluating teachers’ immediate and long-term willingness to continue using online teaching after the pandemic ends, or if further waves (such as newer variants of the virus) of the pandemic require a return to distance learning.

Uniquely, this study evaluates the predictive influence of psychological need thwarting of online teaching, which itself is an emerging construct, from a longitudinal perspective. While the influence of the construct of psychological need thwarting and satisfaction has been simultaneously evaluated by some prior studies, the present study emphasizes the role of PNT in order to (a) address the lack of empirical studies evaluating PNT, as compared to psychological need satisfaction, particularly in the context of online teaching and (b) to avoid the inclusion of too many items in both the questionnaire and model, which would create a burden for respondents while also making validation of the PNTSOT instrument difficult. Some recent studies (Costa et al., 2019; Moè and Katz, 2020; Rodríguez-Meirinhos et al., 2020), have modelled and evaluated the complex interaction among elements of both psychological need thwarting and satisfaction or evaluated profiles including elements of both psychological need thwarting and satisfaction (Warburton et al., 2020), finding the expected positive impact of psychological need satisfaction as compared to frustration. Many of these studies have advocated for longitudinal research (Costa et al., 2019; Rodríguez-Meirinhos et al., 2020; Warburton et al., 2020). Thus, the present study, in the specific context of online teaching, seeks to better understand the role of PNT of online teaching in terms of teachers’ psychological well-being, intention to teach online, and satisfaction with online teaching, utilizing longitudinal data while, simultaneously, adopting a model for testing the immediate and delayed effects of PNT of online teaching.

Furthermore, in the present study, we adopt a research design that integrates cross-sectional and longitudinal elements to test the immediate and delayed effects of PNT of online teaching while simultaneously systematically evaluating the psychometric properties of an instrument for measuring PNT of online teaching (i.e., PNTSOT), originally developed by Yi et al. (2021). In addition to confirming the factorial validity and internal reliability, which have already been sufficiently evaluated by Yi et al. (2021), the core purpose of this study is to address a knowledge gap concerning the lack of longitudinal data required for more rigorous psychometric evaluation, such as test–retest reliability, longitudinal measurement invariance, and predictive validity regarding the association of PNT of online teaching (as measuring during school closure) with other relevant factors, such as future intention to continue online teaching and the long-term impacts on teachers’ psychological well-being (measured when restrictions are removed and offline teaching is available).

## Materials and methods

### Participants

This study was conducted in a city in a province in central China which had implemented mandatory online teaching, with campuses closed due to multiple COVID-19 infections reported at the end of October 2021. It should be noted that although the Chinese government had relaxed COVID-19 restrictions since September 2020 (i.e., indoor activities were allowed and school campuses were reopened), once an infection was reported, restrictive measures were still untaken immediately in order to limit infections. As such, after an outbreak of the pandemic in the city in which the study took place, the city government decided to close all school campuses and fully implement online courses from November 3, 2021. Our research team has been engaged in long-term collaboration with the city’s educational authorities, providing psychological counseling services and regularly holding mental health workshops for the teachers in the city. Thus, in order to monitor the mental health status of teachers during this quarantine period (i.e., 2 weeks after the full implementation of online teaching), our research team, with the assistance of local educational authorities, conducted an online survey of primary and secondary school teachers (Time 1: mid-November, 2021). A follow-up collection of data was conducted after a two-month interval (Time 2: mid-January, 2022). At Time 2, campuses had reopened for 2 weeks and mandatory online teaching was no longer being implemented, with schools returning to a face-to-face mode of instruction. The survey was administered through an online questionnaire, forwarded by the educational administration of each school district to each school in their jurisdiction for voluntary completion by teachers. A total of 9,554 (Time 1) and 4,176 (Time 2) teachers completed the online survey (cross-sectional portion). Participants completing the first survey were asked to leave their email if they would like to participate in a follow-up survey after 2 months. A total of 1,642 school teachers left their email information and participated in the longitudinal portion of the study. Written informed consent was obtained electronically on the first page of the online survey, providing participants with information on the purpose of the research, the affiliation of the researchers, and a guarantee of privacy and anonymity through appropriate storage and curation of the collected data. This study was approved by the Jiangxi Psychological Consultant Association (IRB ref.: JXSXL-2020-J013).

### Measures

The design of the survey was purposefully arranged to minimize the burden to the respondents, providing questions which were relevant only to the current situation (mandatory online teaching due to the recent outbreak). Due to the sudden nature of the announcements related to school closure, teachers were required to conduct online teaching from home and, at this point in time, were required to create, prepare, and manage a great deal of instructional materials. As such, the data collected at Time 1 included measures related to online teaching (including the PNTSOT and a questionnaire assessing satisfaction with online teaching) and a measure of psychological distress (DASS-21).

For both theoretical and practical reasons, a measure of teacher burnout was not included at Time 1, but was evaluated at Time 2. Theoretically, the construct of burnout was utilized in our model as a predicted variable, indicative of the long-term effects of PNT and psychological well-being from a longitudinal perspective, and thus was not included in data collected a Time 1. From a practical perspective, this measure was used only for Time 2 in order to reduce the length of the survey at Time 1 and to avoid influencing teachers’ attitudes towards the longer-term impacts of online teaching, with general attitudes towards online teaching and measures of psychological well-being collected using the PNTSOT and DASS-21 instruments.

For the assessment of satisfaction with online teaching, a single question was posed (Time 1): “How would you rate the effectiveness of your online teaching?” with possible responses varying from very dissatisfied (UNESCO, 2020) to very satisfied (Haug et al., 2022). To evaluate teachers’ intention to continue using online teaching another question was included in the survey for Time 2: “Would you like to continue using online teaching in the future?” with possible responses varying was from very unwilling (UNESCO, 2020) to very willing (Shamir-Inbal and Blau, 2021).

#### Psychological need thwarting scale of online teaching

The PNTSOT developed by Yi et al. (2021) was used to assess the extent of psychological need thwarting during online teaching. As mentioned above, the PNTSOT includes three subscales (i.e., autonomy, competence, and relatedness thwarting) with means and standard deviations for each question by subscale provided in Table 1. The PNTSOT was rated on a seven-point Likert-type scale (ranging from 1 to 7) with higher scores indicating a greater degree of psychological need thwarting during online teaching. The PNTSOT has demonstrated good factorial validity among primary and middle school teachers (Yi et al., 2021). Specifically, the factor structure of the PNTSOT was consistent with the original structure (i.e., a three-factor structure) of the Chinese version of the TPNTS reported in Chen et al. (Chen et al., 2020), where the results of CFA demonstrated acceptable fit according to relevant indices (CFI = 0.97, NNFI = 0.95, RMSEA = 0.09, and SRMR = 0.05). The PNTSOT was administered twice in the present study. For the survey at Time 1, the instructions asked participants to respond while considering their current situation in implementing online teaching, while the instructions for Time 2 asked respondents to recall their experiences in implementing online teaching for the previous 2 months. The aim of this wording was to conduct a more systematic evaluation of the reliability and validity of the PNTSOT through a focus on the collection of longitudinal data which could be evaluated in terms of test–retest reliability. Details on the psychometric characteristics of the PNTSOT are presented in the results section.

**Table 1:** Demographics of the participants and the descriptive statistics of the observed variables.

Source	Yi et al. (12)^a^	The present study (cross-sectional portion)		The present study (longitudinal portion)	
Occasion	2020.5-6	2021.11 (Time 1)	2022.01 (Time 2)	2021.11 (Time 1)	2022.01 (Time 2)
Valid number	9,030	9,554	4,176	1,642	
School type (primary school); *n* (%)	5,838 (64.65%)	6,004 (62.8%)	2,580 (61.78%)	1,159 (70.6%)	
Sex (female); *n* (%)	6,563 (72.7%)	6,933 (72.6%)	3,190 (76.4%)	1,305 (79.5%)	
Age; mean (SD)	33.94 (8.81)	37.20 (9.63)	34.76 (10.04)	34.22 (8.72)	
A. Psychological need thwarting scale of online teaching; mean (SD)					
Subscale of autonomy					
1. In online courses during the pandemic, I cannot decide for myself how I want to teach	3.87 (1.38)	3.69 (1.48)	3.63 (1.64)	3.57 (1.48)	3.42 (1.62)
2. In online teaching work during the pandemic, I feel there is pressure that affects my behavior and requires me to comply in a certain way	4.00 (1.42)	3.96 (1.52)	3.69 (1.67)	3.94 (1.56)	3.54 (1.65)
3. I have to follow a prescribed online teaching style during the pandemic.	4.22 (1.43)	4.33 (1.48)	4.05 (1.62)	4.32 (1.49)	3.90 (1.59)
4. During the pandemic, I feel pressure from the external environment that limited me in choosing a particular online teaching style.	4.01 (1.43)	3.96 (1.49)	3.75 (1.62)	3.86 (1.52)	3.61 (1.58)
Overall mean (autonomy)	4.02 (1.09)	3.98 (1.15)	3.78 (1.37)	3.92 (1.19)	3.62 (1.35)
Subscale of competence					
5. There are some online teaching situations that make me feel incapable in my daily work environment during the pandemic.	4.41 (1.55)	4.45 (1.57)	4.15 (1.73)	4.45 (1.61)	4.07 (1.71)
6. I sometimes talk about the things that make me feel powerless to do my online teaching job during the pandemic.	4.25 (1.49)	4.16 (1.53)	3.96 (1.66)	4.11 (1.58)	3.87 (1.65)
7. Online teaching during the pandemic sometimes makes me feel powerless.	4.24 (1.53)	4.22 (1.56)	3.97 (1.69)	4.16 (1.61)	3.88 (1.68)
8. Due to the lack of training opportunities in my environment, I feel that I am capable of performing online teaching tasks.	3.18 (1.39)	3.09 (1.39)	3.10 (1.48)	3.01 (1.37)	2.95 (1.41)
Overall mean (competence)	4.02 (1.22)	3.97 (1.25)	3.79 (1.42)	3.93 (1.28)	3.69 (1.39)
Subscale of relatedness					
9. I feel disconnected from other colleagues and leaders when teaching online during the pandemic.	2.74 (1.32)	2.69 (1.34)	2.76 (1.42)	2.59 (1.30)	2.61 (1.33)
10. I do not feel that my colleagues and leaders care about me when teaching online during the pandemic.	2.93 (1.42)	2.82 (1.40)	2.89 (1.48)	2.70 (1.38)	2.76 (1.40)
11. I feel that my colleagues and leaders are jealous of me when I achieve good results in online teaching during the pandemic.	2.34 (1.17)	2.26 (1.17)	2.42 (1.31)	2.14 (1.14)	2.32 (1.22)
12. I feel that my colleagues and leaders do not like me when I conduct online teaching during the pandemic.	2.31 (1.16)	2.25 (1.15)	2.41 (1.29)	2.14 (1.13)	2.32 (1.23)
Overall mean (relatedness)	2.58 (1.09)	2.50 (1.10)	2.62 (1.23)	2.39 (1.07)	2.50 (1.16)
B. Psychological distress	15.04 (20.03)	20.15 (20.98)	22.44 (24.42)	19.62 (20.22)	19.12 (21.78)
C. Teacher burnout − emotional exhaustion	Not applicable		3.51 (1.54)	Not applicable	3.46 (1.52)
D. Satisfaction with online teaching (number of participants choosing satisfactory or very satisfactory); *n* (%) – measured at Time 1	Not applicable	6,595 (69.02%)	Not applicable	1,143 (69.61%)	Not applicable
E. Intention of adopting online teaching in the future (number or respondents responding with a score of 6 or higher, on a 10-point scale); *n* (%) – measured at Time 2	Not applicable	Not applicable	1,578 (37.78%)	Not applicable	615 (37.45%)

#### Teacher burnout

To evaluate teacher burnout at Time 2, this study utilized the subscale of “Emotional Exhaustion” from the Chinese version of the Primary and Secondary School Teachers’ Job Burnout Questionnaire (CTJBO). The questionnaire includes 22 items and was developed by Wu et al. (2016) as an adaptation of the Maslach Burnout Inventory (Maslach and Jackson, 1981) revised to fit the cultural and linguistic context of primary and middle school teachers in Mainland China. The CTJBO includes three dimensions: emotional exhaustion (8 items), depersonalization (8 items) and reduced personal accomplishment (6 items; Wu et al., 2016). The CTJBO scale has demonstrated satisfactory factorial validity (Wu et al., 2016) among primary and middle schoolteachers (i.e., RMSEA = 0.06, NFI = 0.950, and CFI = 0.960). Sample items from the Emotional Exhaustion sub-scale of the CTJBO are “I feel exhausted after a long day of work” and “I feel very tired when I wake up in the morning.” The item adopted a Likert-type, ranging from 1 (strongly disagree) to 7 (strongly agree). The internal consistency was high for both longitudinal and cross-sectional data in the present study (Cronbach’s alpha for both was 0.95).

#### Depression, Anxiety, and Stress Scale (DASS-21)

The Chinese version of the 21-item Depression, Anxiety, and Stress Scale (DASS-21) was adopted to measure teachers’ psychological distress during school closure (Time 1) and after restrictions were lifted and face-to-face teaching resumed (Time 2). The survey instructions asked the participants to reflect on their current mental state during school closure (for Time 1) or their mental state in the recent 2 weeks (for Time 2). The DASS-21 is equally divided according to three emotional states: depression, anxiety, and stress (Lovibond and Lovibond, 1996). Items are rated on a four-point Likert-type scale (ranging from 0 to 3) with higher scores reflecting greater levels of these three emotional states. Recently, several studies have provided empirical evidence that the scores of the three subscales reflect general negative emotion (Yeung et al., 2020; Zanon et al., 2020). That is, the average score of all items from the three subscales can serve as an indicator of psychological distress. Moreover, according to Lovibond and Lovibond (1996), the summed score of the subscales multiplied by two can reflect clinical levels of psychological distress when exceeding the following cut-off values: depression (Shamir-Inbal and Blau, 2021), anxiety (Munoz-Najar et al., 2021), and stress (Bank, 2021). The DASS-21 has been translated into Chinese and has been widely used with Chinese samples, including studies by Chan et al. (2012) and Wang et al. (2015). In Chan et al.’s (2012) study with typical adults, the DASS-21 demonstrated satisfactory factorial validity (i.e., RMSEA = 0.08; SRMR = 0.06; CFI = 0.98; and NNFI = 0.97). In this present study, the Cronbach’s alpha of the overall DASS-21 was 0.95 (Time 1) and 0.96 (Time 2) using the longitudinal data.

### Data analysis

In order to thoroughly and systematically evaluate the impact of PNT of online teaching in terms of immediate effects (including psychological distress and satisfaction with online teaching) and delayed effects (including intention to continue online teaching and burnout), mean values and correlation coefficients for all variables were first analyzed. Subsequently, the reliability and factorial validity of PNTSOT were evaluated. Finally, structural equation modelling (SEM) was utilized in order to test the causal relationships among variables, including the effect of PNT of online teaching on psychological distress and satisfaction with online teaching (both measured at Time 1), as well as the delayed effects on intention to continue online teaching and burnout (both measured at Time 2). Furthermore, in order to evaluate the psychometric properties of the PNTSOT, data collected by Yi et al. (2021) was also included in the present study for analysis (i.e., descriptive statistics for the variables, tests of model fit, and measurement invariance). The procedures for data analysis conducted in this study are described in detail below.

Descriptive statistics were first used to analyze the characteristics of the participants and their responses on the PNTSOT and criterion variables (i.e., burnout, satisfaction with online teaching, intention to continue online teaching in the future, and psychological distress). Moreover, Pearson correlations among the observed variables (averaged values) for the PNTSOT, burnout, satisfaction of the online teaching, intention of continuing online teaching in the future, and psychological distress were computed for the longitudinal data. Following, McDonald’s ω, the Intraclass correlation coefficient (ICC) with a 2-way mixed effects model combining a Bland–Altman plot, and CFA were used to evaluate internal reliability, test–retest reliability, and factorial validity. It should be noted that, during the development of the Chinese version of TPNTS, on which PNTSOT is based, item 8 (“due to the lack of training opportunities in the environment, I feel that I am not competent in my daily work tasks”) was found to be cross-loaded for both the relatedness thwarting and competence thwarting factors. Thus, as the inclusion of item 8 may affect the overall measurement quality of the scale (Chen et al., 2020), we conducted CFA to evaluate whether this item should be included in the PNTSOT based on the data collected by our current study.

**Figure 1:**
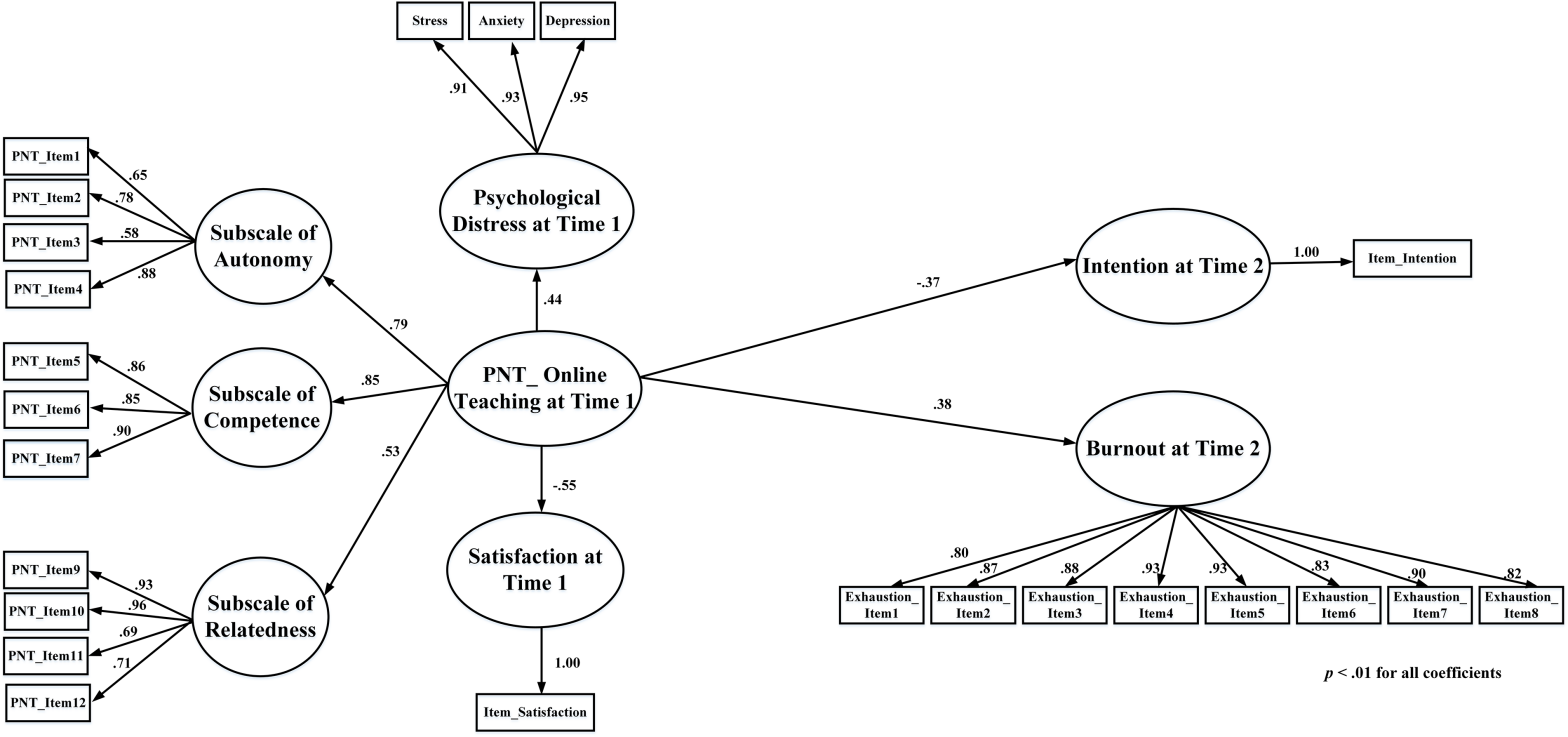
Criterion validity as examined by structural equation modeling (SEM; including second-order confirmatory factor analysis and a causal model; *n* = 1,642); variables include psychological need thwarting of online teaching (PNT_Online Teaching), satisfaction with online teaching (Satisfaction), and intention of continuing online teaching the future (Intention).

After scrutinizing the factorial validity of each sample, a multi-group and longitudinal invariance test was conducted to assess whether the PNTSOT possessed measurement invariance across different occasions. Finally, we constructed and tested a structural equation model (SEM) including a higher-order CFA of PNTSOT and a causal model to test criterion validity (see Figure 1). Specifically, in this model, the higher-order latent variable of PNTSOT (Time 1) served as an exogenous variable (with its three subscales serving as first order latent variables), with psychological distress (Time 1 and Time 2), satisfaction with online teaching (Time 1), intention for future online teaching (Time 2), and teacher burnout (Time 2) added as endogenous variables.

Due to violation of the assumption of a normal distribution for the longitudinal data from the PNTSOT (the values of Shapiro–Wilk test were 0.99 and 0.97, both *p* < 0.01), estimation utilizing diagonally weighted least squares (DWLS) was adopted for CFA, tests of measurement invariance, and SEM, as DWLS is more suitable for dealing with non-normally distributed data (Li, 2016). In terms of the evaluation of factorial and criterion validity, we adopted the following indices: Satorra-Bentler Scaled Chi-Square (SB χ^2^), comparative fit index (CFI), non-normed fit index (NNFI), root mean square error of approximation (RMSEA), and standardized root mean square residual (SRMR). CFI and NNFI values of 0.95 or higher, RMSEA values of 0.06 or lower, and SRMR values of 0.08 or lower were considered acceptable (Hu and Bentler, 1999).

Finally, a series of model comparisons were conducted to evaluate the measurement equivalence of the PNTSOT across different occasions. More specifically, the following comparisons were made: (a) the configural model (i.e., baseline model) was compared with the factor-loading constrained equal model; (b) the factor-loading constrained equal model (a less constrained model) was compared with the factor-loading and item intercept constrained equal model (a more constrained model); (c) the factor-loading and item intercept constrained equal model (a less constrained model) was compared with the factor-loading, item intercept, and errors constrained equal model (a more constrained model); (d) the factor-loading and item intercept constrained equal model (a less constrained model) was compared with the factor-loading, item intercept, and factor variance, as well as the covariance constrained equal model (a more constrained model). The differences in CFI, RMSEA, and SRMR from the less constrained models to the more constrained model were used to judge whether or not measurement invariance was supported: ΔCFI > −0.01, ΔRMSEA <0.015, and ΔSRMR <0.03 (for factor loading) or ΔSRMR <0.01 (Chen, 2007).

## Results

### Participant characteristics, observed means, and relationships among variables

Table 1 presents the characteristics of the participants from data collected from the two cross-sectional surveys and the longitudinal study with mean observed scores for the variables of interest in this study. In terms of demographic characteristics, the participants in this study were mostly from primary schools (61.7–70.6%), mostly female (72.6–79.5%), with an average age between 34 and 37 years, which are similar to the participant characteristics reported by Yi et al. (2021). The above demographic variables were also close to the overall population statistics [i.e., from all primary and middle schoolteachers in mainland China; 2020 Education Statistics (Internet), 2021], in terms of age (i.e., population mean age of 37.78), school type (64% of schools nation-wide are primary schools), and gender (70% of teachers are female) which supports the representativeness of the participants. Supplementary Table 1 also reports the Pearson correlations among the observed variables of the three subscales of the PNTSOT.

Next, in order to evaluate potential differences between primary and middle school teachers, we evaluated the mean scores on all variables. The results of independent sample *t*-tests demonstrated very few significant differences (with trivial effect sizes) between primary and middle school teachers on the variables of interest (see Supplementary Table 2). Moreover, the results of CFA and measurement invariance also confirmed that PNTSOT is appropriate for use with both groups of teachers with no bias (see Supplementary Table 3). Consequently, it was not necessary to separately evaluate the two groups of teachers in the analysis and the data from both groups was pooled for subsequent analyses.

For the purpose of evaluating the relationships among variables, including the predictive role of PNT of online teaching on the delayed measures of intention to continue online teaching and burnout, PNTSOT scores from Time 1 were utilized. However, for the purpose of validation, differences in responses to the PNTSOT instrument were evaluated from Time 1 (assessing reported PNT of online teaching at that time) and Time 2 (where respondents were asked to reflect on their previous 2 months of online teaching). Additionally, differences in perception between Time 1 and Time 2 were evaluated in order to better interpret the effects of PNT of online teaching in the context of other variables of interest.

Among the three dimensions of psychological need thwarting, autonomy and competence thwarting were higher during Time 1 (as compared to Time 2), with mean autonomy thwarting of 3.98 (cross-sectional data) and 3.92 (longitudinal data) and mean competence thwarting of 3.97 (cross-sectional data) and 3.93 (longitudinal data). The scores of these two subscales decreased when returning to offline instruction (Time 2) for both the cross-sectional and longitudinal data, with means for autonomy thwarting of 3.78 (cross-sectional data) and 3.62 (longitudinal data), and means for competence thwarting of 3.79 (cross-sectional data) and 3.69 (longitudinal data). Interestingly, relatedness thwarting increased from Time 1 to Time 2, for both cross-sectional and longitudinal data; with mean values at Time 1 of 2.50 (cross-sectional data) and 2.39 (longitudinal data); and mean values at Time 2 of 2.62 (cross-sectional data) and 2.50 (longitudinal data). It is noted that despite changes in the observed scores for PNTSOT over time, tests for longitudinal reliability and validity are still necessary to evaluate ICC and longitudinal measurement invariance.

The participants’ overall psychological distress was 20.15 (cross-sectional data) and 19.62 (longitudinal data) at Time 1, while psychological distress scores were 22.44 (cross-sectional data) and 19.12 (longitudinal data) at Time 2. Regarding specific emotional states, the percentage of teachers with clinical depression increased in both cross-sectional (from 25.2 to 29.9%) and longitudinal data (from 22.41 to 23.51%); the percentage of anxiety and stress increased in the cross-sectional data (anxiety: from 35.5 to 40.35%; stress: from 15.7 to 19.0%) but decreased in the longitudinal study (anxiety: from 35.87 to 34.71%; stress: from 16.13 to 15.71%). Participants’ burnout was considered moderate (cross-sectional data: 3.51; longitudinal data: 3.46) due to the value being close to the median of the scale (i.e., 3.50). Finally, more than half of the teachers were satisfied or very satisfied with their online teaching, as indicated by responses of either “satisfied” (3 out of 4 on the Likert-type scale) or “very satisfied” (4 out of 4 on the Likert-type scale); with 69.02% satisfaction based on the cross-sectional data and 69.61% based on the longitudinal data. However, only 37% of the participants responded they would like to continue using online teaching in the future (i.e., respondents who selected a value of 6 or more out of a total of 10).

Table 2 displays the Pearson correlations among variables of interest for the longitudinal data. PNT of online teaching (for both Time 1 and Time 2) was significantly correlated with all criterion variables. Specifically, a negative correlation was found between PNT of online teaching and satisfaction at Time 1 (*r =* −0.39; *p* < 0.001) and Time 2 (*r =* 0.28; *p* < 0.001); and a negative correlation between PNT of online teaching and intention to teach online found at Time 1 (*r =* −0.28; *p* < 0.001) and Time 2 (*r =* −0.26; *p* < 0.001). Moreover, PNT of online teaching was significantly and positively correlated with burnout and psychological distress, with correlation coefficients ranging from 0.27 to 0.44 (all *p* < 0.001). These observed associations were supported by the results of structural equation modelling (Figure 1), with the model demonstrating significant causal paths in line with the correlations reported here. These findings are provided in the following sub-section.

**Table 2:** Pearson correlations among the variables of psychological need thwarting (PNT) of online teaching, satisfaction, intention, burnout, and psychological distress for the longitudinal data (*n* = 1,642).

	1	2	3	4	5	6	7
1. PNT of online teaching (Time 1)	1						
2. PNT of online teaching (Time 2)	0.56^**^	1					
3. Satisfaction (Time 1)	−0.39^**^	−0.28^**^	1				
4. Intention (Time 2)	−0.28^**^	−0.26^**^	0.27^**^	1			
5. Burnout (Time 2)	0.27^**^	0.44^**^	−0.20^**^	−0.15^**^	1		
6. Psychological distress (Time 1)	0.36^**^	0.32^**^	−0.20^**^	−0.11^**^	0.36^**^	1	
7. Psychological distress (Time 2)	0.27^**^	0.42^**^	−0.15^**^	−0.06^*^	0.52^**^	0.58^**^	1

Time 1 was measured at mid-November, 2021, Time 2 was measured at mid-January, 2022; ^**^p < 0.01, ^*^p < 0.05.

### Reliability and validity of the PNTSOT

**Figure 2:**
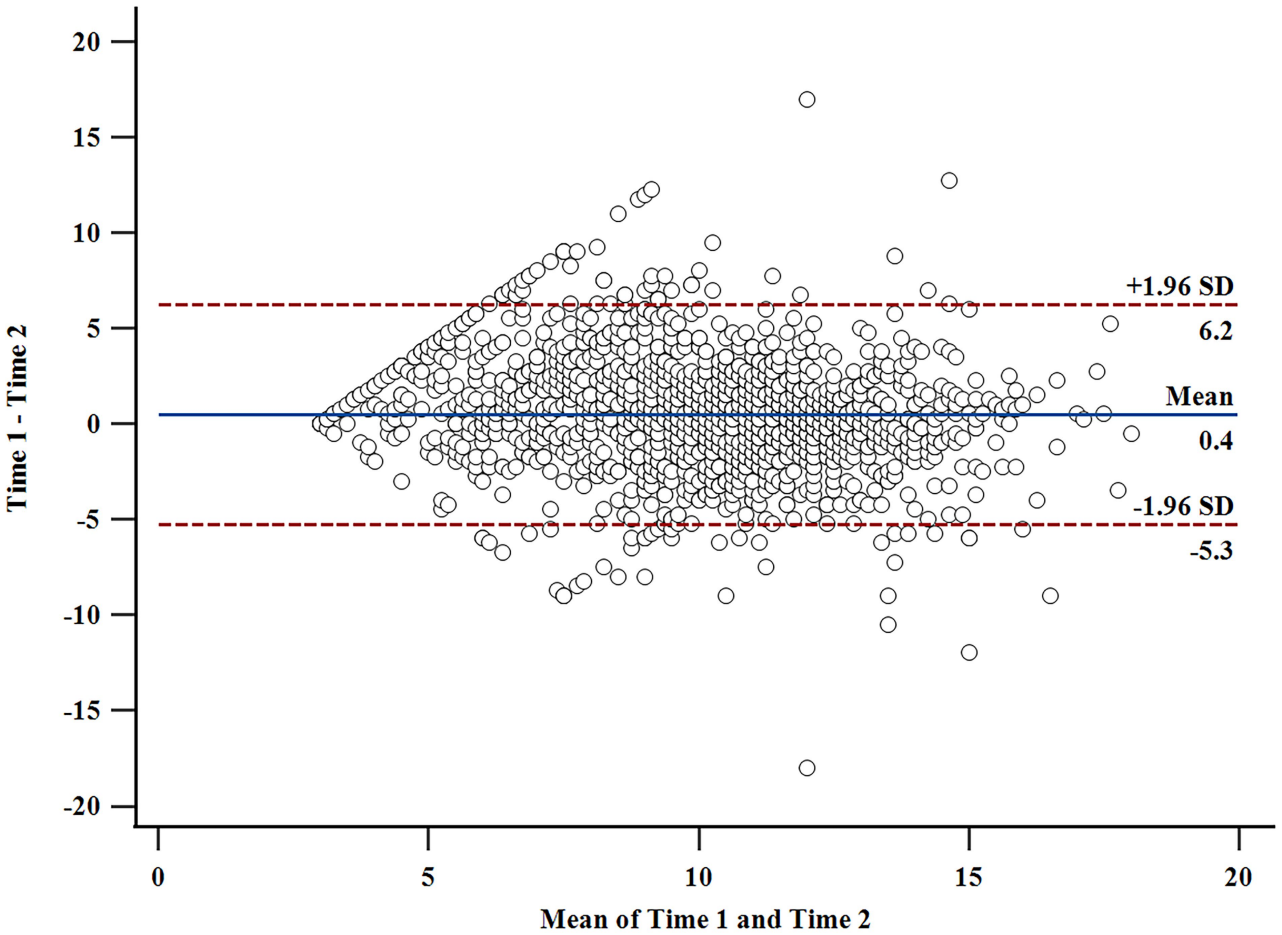
Bland–Altman plot of the Psychological Need Thwarting Scale of Online Teaching (PNTSOT) from Time 1 and Time 2 (*n* = 1,642).

The internal reliability of the PNTSOT was satisfactory, given that the McDonald’s ω of three subscales were all higher than 0.80 for longitudinal participants (Time 1: Autonomy thwarting *ω* = 0.80, Competence thwarting *ω* = 0.87, and Relatedness thwarting *ω* = 0.88; Time 2: Autonomy thwarting *ω* = 0.86, Competence thwarting *ω* = 0.90, and Relatedness thwarting *ω* = 0.91). Similar findings were reported for the cross-sectional data, with coefficients exactly the same as with the longitudinal data (with the exception of an *ω* value of 0.78 for autonomy thwarting at Time 1). Moreover, the ICC for PNTSOT using a 2-way mixed effects model was 0.71, indicating acceptable test–retest reliability across the two-month interval. Analysis also demonstrated that 95% of the data points lied within ±1.96 SD of the mean difference in a Bland–Altman plot (see Figure 2), with only 88 data points outside of that range, suggesting appropriate consistency of PNTSOT between the two occasions.

The results of CFA demonstrated that an 11-item version of PNTSOT (i.e., excluding item 8) had a better model fit than the scale with 12 items (see Table 3). Specifically, in the 11-item version, CFI and NNFI ranged from 0.990 to 0.999, and RMSEA and SRMR ranged from 0.017 to 0.068, showing good fit. Subsequently, we further tested the measurement invariance of the PNTSOT (see Table 4). The results indicated that the 11-item PNTSOT demonstrated scalar invariance (i.e., with loadings and thresholds equal) across different occasions. Namely, the comparisons of pairs of data points collected in our present study matches the findings of Yi et al. (2021). However the 12-item version, which included item 8, failed to demonstrate scalar invariance. Furthermore, when online teaching was being launched (i.e., in the study of Yi et al. (2021) and at Time 1 of our study), the PNTSOT achieved the strictest measurement invariance, namely having error variance equivalence for each item (i.e., the measurement error of each item was equal across different occasions) and also had the same factor variance as well as covariance (i.e., the variance of the three factors and the covariance between factors in PNTSOT was equal across different occasions). However, for Time 2 of our study, the PNTSOT did not pass the measurement equivalence criteria between occasions (i.e., a comparison between the data from Yi et al. (2021) and Time 2 of our study and a comparison between Time 1 and Time 2 of our study) in terms of measurement error variance, factor variance, and factor covariance. However, this result must be interpreted with caution, since the instructions for the survey at Time 2 asked participants to consider their situation 2 months earlier, when online teaching was launched, which required recalling their memory of online teaching at that time.

**Table 3:** Results of model fit.

	*χ^2^* (*df*)	CFI	NNFI	RMSEA (90% confidence interval)	SRMR
Yi et al. (2021) (12 items)	1795.06 (51)	0.983	0.978	0.062 (0.059–0.064)	0.088
Yi et al. (2021) (11items)	573.55 (41)	0.990	0.990	0.038 (0.035–0.041)	0.055
Time 1^a^ (12 items)	594.64 (51)	0.996	0.994	0.033 (0.031–0.036)	0.089
Time 1^a^ (11 items)	156.43 (41)	0.999	0.999	0.017 (0.014–0.020)	0.055
Time 2^a^ (12 items)	1478.51 (51)	0.983	0.978	0.082 (0.078–0.085)	0.076
Time 2^a^ (11 items)	431.05 (41)	0.990	0.990	0.048 (0.044–0.052)	0.046
Time 1^b^ (12 items)	521.87 (51)	0.979	0.973	0.075 (0.069–0.081)	0.089
Time 1^b^ (11 items)	166.95 (41)	0.990	0.990	0.043 (0.037–0.050)	0.060
Time 2^b^ (12 items)	952.77 (51)	0.974	0.966	0.104 (0.098–0.110)	0.079
Time 2^b^ (11 items)	356.12 (41)	0.990	0.990	0.068 (0.062–0.075)	0.047
Model of testing criterion validity (12 items)	1596.43 (268)	0.981	0.978	0.055 (0.052–0.058)	0.084
Model of testing criterion validity (11 items)	326.25 (245)	0.999	0.999	0.014 (0.009–0.018)	0.071

a Cross-sectional data;

b Longitudinal data. CFI, comparative fit index; NNFI, nonnormed fit index; RMSEA, root mean square error of approximation; SRMR, standardized root mean square residual.

**Table 4:** Tests of measurement invariance.

	Configural model		Loadings constrained as equal		Loadings and thresholds constrained as equal		Loadings, thresholds, and errors constrained as equal		Loadings, thresholds, factor variance, and covariance constrained as equal^a^	
Yi et al. (2021) and Time 1										
	12 items	11 items	12 items	11 items	12 items	11 items	12 items	11 items	12 items	11 items
*Χ*^2^ (*df*) or *ΔΧ*^2^ (*Δdf*)	1863.04 (102)	571.89 (82)	−57.32 (9)	−19.72 (8)	151.77 (9)	163.32 (8)	115.12 (12)	95.92 (11)	−280.42 (6)	**−**87.78 (Gouëdard et al., 2020)
CFI or ΔCFI	0.970	0.990	**0.001**	**0.001**	**−0.002**	**−0.003**	**−0.002**	**−0.002**	**0.005**	**0.001**
RMSEA or ΔRMSEA	0.043	0.025	**−0.002**	**−0.001**	**0.000**	**0.002**	**−0.001**	**0**	**−0.005**	**−0.003**
SRMR or ΔSRMR	0.050	0.035	**0.000**	**0.001**	**−0.038**	**−0.024**	**0.001**	**0.001**	**0.004**	**0.004**
Yi et al. (2021) and Time 2										
*Χ*^2^ (*df*) or *ΔΧ*^2^ (*Δdf*)	4479.91 (102)	1439.87 (82)	107.75 (9)	**−**9.29 (Munoz-Najar et al., 2021)	−208.01 (9)	117.47 (Munoz-Najar et al., 2021)	1779.46 (12)	1610.66 (11)	−82.94 (6)	329.05 (Gouëdard et al., 2020)
CFI or ΔCFI	0.896	0.961	−0.002	**0.001**	0.005	**−0.003**	−0.042	−0.046	0.002	**−0.009**
RMSEA or ΔRMSEA	0.081	0.050	−0.003	**−0.002**	−0.005	**−0.001**	0.01	0.018	−0.002	**0.004**
SRMR or ΔSRMR	0.036	0.023	0.002	**0.001**	−0.021	−**0.001**	0.004	**0.004**	0.0231	0.027
Time 1 and Time 2										
*Χ*^2^ (*df*) or *ΔΧ*^2^ (*Δdf*)	2614.71 (225)	1065.26 (183)	−38.16 (9)	−22.12 (8)	99.07 (9)	216.75 (Munoz-Najar et al., 2021)	382.94 (Yi et al., 2021)	295.24 (11)	156.15 (6)	454.16 (16)
CFI or ΔCFI	0.875	0.943	0.003	**0.002**	−0.005	−**0.010**	−0.019	−0.019	−0.008	−0.029
RMSEA or ΔRMSEA	0.081	0.054	−0.003	−**0.002**	0	**0.005**	0.004	**0.005**	0.002	**0.010**
SRMR or ΔSRMR	0.038	0.027	0	**0**	−0.020	−**0.011**	0.001	**0.001**	0.013	0.015

a The change was calculated with the model of Loadings and Thresholds Constrained as Equal. CFI, comparative fit index; RMSEA, root mean square error of approximation; SRMR, standardized root mean square residual. Supported measurement invariance values are in bold (i.e., ΔCFI > −0.01; ΔRMSEA <0.015; ΔSRMR <0.03 (for factor loading) or ΔSRMR <0.01) (for item threshold).

Regarding criterion validity, concurrent and predictive validity were assessed by SEM (see Figure 1). Since the residual was too high when the scores for DASS-21 at Time 1 and Time 2 were both placed in the model simultaneously, ultimately only the scores for psychological distress at Time 1 were included for SEM. As the model demonstrated an acceptable fit (see Table 3), we further scrutinized the path coefficients of the model. The results supported both concurrent and predictive validity, since the PNT of online teaching at Time 1 was significant and positively associated with psychological distress at Time 1 (*γ* = 0.44, *t* = 7.45, *p* < 0.001), negatively associated with the satisfaction of online teaching at Time 1 (*γ* = −0.55, *t* = −19.95, *p* < 0.001), negatively associated with the intention to continue online teaching at Time 2 (*γ* = −0.37, *t* = −11.45, *p* < 0.001), and positively associated with burnout at Time 2 (*γ* = 0.38, *t* = 12.39, *p* < 0.001).

## Discussion

### Purpose and main findings

Recent literature on the effects of the COVID-19 pandemic on teachers has widely reported low levels of satisfaction with online teaching (Fauzi and Khusuma, 2020), an unwillingness to use online teaching in the future (Zheng and Song, 2020), and even poor psychological well-being (Aperribai et al., 2020; Richmond et al., 2020; Palma-Vasquez et al., 2021; Silva et al., 2021; Jelińska and Paradowski, 2021a,b). As such, the purpose of the present study was two-fold. First, the relationship between PNT of online teaching (characterized by the thwarting of teachers’ autonomy, competence, and relatedness needs) and concurrent satisfaction with online teaching and psychological distress, as well as the predictive effect of PNT of online teaching on intention to continue online teaching after a return to regular classes and teacher burnout 2 months later. In terms of the first aim, the study found that PNT of online teaching is significantly and negatively associated with satisfaction with online teaching, such that increased need thwarting lowers teachers’ satisfaction. At the same time, PNT of online teaching is positively associated with teachers’ psychological distress, such that increased need thwarting is associated with increased depression, anxiety, and stress. Furthermore, need thwarting caused by online teaching is predictive of teacher burnout after 2 months as well as a decrease in teachers’ intention to use online teaching when no longer required to do so. These findings extend those of previous studies – wherein psychological need thwarting, generally, have been shown to significantly and negatively impact teachers’ psychological well-being (Cuevas et al., 2015; Chen et al., 2020) – by assessing delayed effects through the analysis of longitudinal data. The second aim was to validate an instrument for evaluating PNT of online teaching, the PNTSOT, which is proposed as a valuable measure for predicting teacher psychological and behavioral outcomes. The results of the present study convincingly support the internal reliability, test–retest reliability, measurement invariance, and criterion validity (concurrent and predictive) of the instrument, based on both cross-sectional and longitudinal data, suggesting that the PNTSOT instrument can be of value for both researchers and practitioners.

### Theoretical implications

#### The lasting effect of PNT of online teaching

In terms of a theoretical contribution, although a few studies have reported a negative relationship between PNT of online teaching and the psychological well-being outcomes using cross-sectional data (Yi et al., 2021), to our knowledge, there have been no studies examining the long-term and delayed effects of mandatory online teaching on teachers’ psychological well-being and intention to utilize online teaching during times when traditional face-to-face instruction can be adopted. Moreover, there has been a general lack of longitudinal data to support the impact of PNT, overall, on outcomes related to individual well-being (Cuevas et al., 2015; Chen et al., 2020; Lagios et al., 2022). Therefore, our results bridge several existing gaps in terms of both theory and practice. Moreover, it should be noted that the effect (causal path) of PNT from online teaching at Time 1 on burnout at Time 2 was Gamma = 0.38, which is higher than effect reported by Huyghebaert et al. (2018) – one of the very few longitudinal studies in this field. In their study, prior PNT stemming from an individual’s workplace had a significant effect on nurses’ burnout 3 months later, with an effect of only 0.12. This difference may reflect the serious manner in which mandatory online teaching deeply frustrated teachers’ psychological needs, resulting in an even more intense and lasting effect on teachers, as a particularly vulnerable population. As such, more longitudinal studies, related to the effects of PNT during mandatory job characteristics during pandemics, are needed to further examine the nature of the effect of PNT across different populations, including how PNT predicts and contributes to burnout or psychological distress. Given the ongoing challenges of the pandemic, and the likelihood of future waves of COVID, including newer variants, the application of the approach outlined in this study can yield important findings into the mechanisms behind the effects of pandemics on individuals in their workplace, including factors relevant to psychological needs and well-being (including distress), and intentions to continue work or, conversely, the potential for burnout.

#### Evaluating changes in PNT of online teaching

Given that measurement equivalence (scalar invariance) was supported for PNTSOT across different occasions, the values from the scale between two points in time (occasions) can be meaningfully compared and interpreted as reflecting a real change in the thwarting of psychological needs. Our results demonstrated that the three components of PNT of online teaching varied with context, depending on the conditions caused by the pandemic and the actions taken by local educational authorities. It is, therefore, not surprising that teachers’ psychological need thwarting stemmed mainly from a stressful environment (Bartholomew et al., 2014; Franco et al., 2022), although the instructions provided in the administration of the PNTSOT asked the participants to respond based on the current situation (Time 1) when campuses were closed and mandatory online teaching was launched. However, given the need to establish test–retest reliability, this potential weakness is also a strength, in terms of the psychometric validation of the instrument. Nevertheless, although the instructions were intentionally worded in order to evaluate perceptions of the same experience (Time 1: mandatory online teaching) between occasions (i.e., for establishing longitudinal measurement invariance), differences were still found between the two occasions. We speculate that this may be due to the influence of changing circumstances (i.e., from mandatory online teaching to face-to-face teaching) on teachers’ perceptions of previous experiences of psychological need thwarting related to online teaching. The existence of this unconscious influence, resulting in an evolving perception over time, is reasonable, as teachers’ current environment (Time 2) resulted in less thwarting of their psychological needs for autonomy and competence, given the use of traditional instruction, thus softening their impressions of the negative feelings of thwarting regarding these two needs during the online teaching period (Time 1).

Unexpectedly, although the closure of schools reduced teachers’ access to job-related resources, it also seemed to have reduced some demanding aspects of the teaching job. These findings highlight the double-edged nature of the teaching profession (Neves de Jesus and Lens, 2005). On the one hand, frequent and direct interaction with students constitutes a major motivator and source of job satisfaction for many teachers (Watt et al., 2012; Benita et al., 2019). On the other hand, the interpersonal and social nature of teaching can also serve as an occupational demand resulting in diminished well-being outcomes (Hilger et al., 2021) upon returning to an offline learning environment, as teachers’ perceptions of thwarting in terms of relatedness (or interpersonal relationships during the online teaching period) were shown to have increased. This suggests that relatedness thwarting contributed more to the overall thwarting of psychological needs from Time 1 to Time 2. One possible reason is that although quarantine restrictions limited connections among teachers, these restrictions may also have reduced the frequency of negative interactions which could arise in the school or workplace. As such, in the early days of returning to the school campus, a certain amount of time to adapt and re-establish healthy interactions may be required. This effect has been noted in studies on return-to-work (Shaw et al., 2020). For example, there is a great deal of work for teachers to complete when they return to campus, including the enforcement of new, strict bio-safety protocols which can increase workload when teachers return to face-to-face environments (Silva et al., 2021). Moreover, increased requirements for group work may also affect teachers’ perception of interpersonal relationships which can subconsciously influence their perceptions of previous online teaching experiences, wherein less group work was required. This result is consistent with the arguments mentioned by Yi et al. (2021) who stated that “lack of interaction with colleagues might have served as a buffer to reduce potential thwarting of relatedness needs due to less potentially negative interactions with colleagues or leaders.” Furthermore, relatedness thwarting was more strongly correlated with the other two forms of PNT when schoolteachers returned to school (Time 2) as compared to during campus closure (Time 1), as is illustrated in Supplementary Table 1. We believe this finding also provides evidence to support the increased influence of relatedness thwarting on overall psychological distress, which can further and explain the unequal longitudinal measurement in terms of the constraint of variance and covariance of factors.

#### The measurement of PNT

In terms of the literature related to the measurement of PNT, starting from the initial assessment of athletes (Bartholomew et al., 2011), an increasing number of studies and contexts have measured PNT, among adolescents populations (Hein et al., 2015), business environments (Lagios et al., 2022), nurses (Huyghebaert et al., 2018), as well as schoolteachers (Cuevas et al., 2015; Chen et al., 2020). Recently, in addressing the context of the COVID-19 pandemic, the focus of PNT was on the emergence of a specific demanding and unpredicted task, online teaching (Yi et al., 2021). In fact, among all contexts used for the evaluation of PNT, longitudinal designs have rarely been adopted. Therefore, our study primarily aimed to further evaluate the longitudinal reliability and validity of the PNTSOT instrument and, in this way, contribute to our understanding of the assessment of PNT which can be applied to different contexts. As such establishing the validity and reliability of the PNT instrument is fundamental to research and practice related to this emerging area of research, as compared to the emphasis prior research has largely placed on psychological need satisfaction (Huyghebaert et al., 2018; Wu et al., 2018; Lagios et al., 2022). In terms of the evaluation of the instrument, it should be noted that we found that item 8 in the competence thwarting subscale negatively affected overall measurement quality, not only making the 12-item version inferior to the 11-item version according each fit index, but also causing the 12-item version to fail to meet the criteria of measurement invariance. This result is consistent with findings related to the instrument as evaluated by Chen et al. (Chen et al., 2020), where item 8 lowered the quality of Chinese version of TPNTS.

### Practical implications

The lasting harm from PNT of online teaching was demonstrated in this study, predicting teachers’ willingness to continue using online teaching as well as the degree of burnout after a period of 2 months. These results highlight the delayed and long-term effect of mandatory online teaching which lasts beyond the period when online teaching is implemented – a finding which has been described by some studies (Kulikowski et al., 2021; Silva et al., 2021; Jelińska and Paradowski, 2021a,b). This finding is a warning that teachers’ psychological need thwarting during online teaching is an issue of significance in terms of long-term psychological, affective, and intentional outcomes. Benefitting from the longitudinal design adopted in this study, we are able to suggest that school administrators provide teachers with effective relief of PNT during online teaching at different occasions. In terms of the three psychological needs that are potentially thwarted by online teaching, relatedness, autonomy, and competence are all relevant to teachers’ psychological well-being and intention to continue online teaching. For example, in addition to the fact that teachers’ need for relatedness may be thwarted due to home isolation, mandatory online teaching can further frustrate teachers’ psychological needs of autonomy and competence.

In terms of competence, most teachers have not received sufficient training or experience in implementing online teaching (Trust and Whalen, 2021; Yi et al., 2021) which naturally leads to a higher perceived barrier related to technology use. This lack of technological pedagogical content knowledge also leads to teachers’ frustration and loss of competence in respect to online teaching tasks. More specifically, it is more critical to address competence thwarting when online teaching, or other initiatives, are immediately enacted. In addition to providing training in technology-integrated instruction, as suggested by several studies as a means to increase teachers’ experience with online teaching (Silva et al., 2021; Trust and Whalen, 2021), in order to address competence thwarting, school leaders should also be more flexible and lenient in their management, allowing more tolerance and flexibility to address (and avoid thwarting) teachers’ autonomy needs during this period of uncertainty. In fact, autonomy is often considered a prerequisite for the development of competence, with scholars emphasizing the importance of “mastery experiences” in order to develop a sense of self-efficacy (Hagger et al., 2020). Mastery, in the context of the pandemic and online teaching involves several key factors, including conceptual technological pedagogical knowledge (TPK), actively engaging in tutorials to enhance digital competencies, and taking the initiative to embrace opportunities to learn (König et al., 2020). In fact, the role of TPK self-efficacy, in particular, during remote (online) teaching during the pandemic has been associated with perceived confidence as well as willingness to continue teaching online (Cahapay and Anoba, 2021) To avoid competence thwarting, and develop these key skills for developing mastery, school leadership must provide unique and complementary resources to help teachers in conducting online teaching, and most importantly, these resources should be provided continuously rather than as a short-term respond to the initial crisis (Matthews et al., 2022).

In terms of autonomy, in many cases teachers were required to use their school’s designated platforms (including software) and follow prescribed course activities (including assessment methods) for online teaching which can result in perceived lack of autonomy in terms their teaching (Kulikowski et al., 2021). As stated in Kulikowski et al. (2021), restrictive rules and standards regarding online teaching methods harm teachers’ autonomy. Thus, in order to address autonomy thwarting, school leaders should be more flexible and lenient in their management, allowing more tolerance and flexibility to address (and avoid thwarting) teachers’ autonomy needs during this period of uncertainty, particularly as teachers voice their needs for receiving more flexibility from the administrators, such as relaxing state standards for curriculum content, flexibility with deadlines, and loosing requirements for evaluation during times of challenge (Chan et al., 2021). While organizations, such as the OECD, suggest setting school-based goals for promoting teacher autonomy, such as professional development on strategies for assisting teachers and parents in working together to implement online learning more smoothly, and the creation of professional communities of learning focused on promoting teacher autonomy (Benita et al., 2019), the cultural context must also be considered. In the specific context of Chinese education, researchers have emphasized the potential thwarting role of specific school-level policies focused on extrinsic goal framing and a “controlling” approach in terms of student and teacher autonomy, requiring change at the organizational level (Yu et al., 2018), advocating for an awareness of the autonomy thwarting that exists within a controlling hierarchical system. As such, school administrators in Chinese schools must balance teachers’ need for support with a climate that promotes voluntary participation and avoids conveying a sense of control over teachers or a reliance on extrinsic goals and rewards. Research on professional development for teachers has highlighted the importance of providing a rationale for teachers and accepting resistance, rather than forcing participation, giving teachers a chance to develop autonomy through hands-on learning supported by a warm and respectful environment in which positive feedback is provided (Aelterman et al., 2016). In sum, mitigating the risk of autonomy thwarting involves offering voluntary, flexible, and respectful opportunities to participate in communities of practice (Thornton, 2021).

From the point of view of relatedness – some literature has reported a separate spike in psychological distress among schoolteachers once schools reopen (72, 73) – the present study provides insights into the interpretation of this situation in terms of teachers’ relatedness thwarting. Given the fact that teachers’ perceptions of the PNT of online teaching were less severe when asked to evaluate online teaching in retrospect provides some hope of a “rebound” effect from the PNT of online teaching or negative experiences with other types of educational technologies, if teachers’ psychological need thwarting can be averted. For example, there is potential in providing more opportunities for preventing relatedness thwarting through the establishing of communities of practice, simultaneously mitigating threats to competence and autonomy through targeted professional development that emphasizes not only skills and knowledge related to new technologies, but also takes into consideration potential thwarting of teachers’ psychological needs. As such, the emotional care provided by school leaders is important during the early days of campus reopening. This kind of emotional care is characterized as warm and empathetic, led and modelled by front-line leaders, rather than enacted by means of an authoritarian style of leadership (Matthews et al., 2022). As such, avoiding thwarting of relatedness, and consequent turnover intention, involves leaders expressing their care for teachers and assisting teachers in maintaining balance of work and family responsibilities. From the experience of school leaders in the United Kingdom during the pandemic, several key leadership strategies were found in terms of relatedness: mitigating external pressures and expectation, adopting adaptive leadership, providing emotional guidance, working to build relationships, and maintaining resilience in an ever-changing and uncertain environment (Beauchamp et al., 2021). Thus, to avoid thwarting of teachers’ relatedness needs, school leaders are encouraged to actively serve as models, maintaining relationships with teachers, parents, and students, assisting the most vulnerable, and adapting models of leadership based on present needs.

In light of the potential for thwarting of competency, autonomy, and relatedness needs, a recurring theme is the importance of professional development. Given the importance of teachers’ psychological needs during challenging times, such as mandatory online teaching, the role of teacher training must also be considered. During teacher training, pre-service teachers can benefit from increased choice and freedom in pursuing individual goals (autonomy), positive feedback through coaching and mentorship which encourages student teachers to identify their unique personal qualities and incorporate these into their teaching (competence), and fostering a sense of the social environment of teaching with attention to individual students (relatedness; Evelein et al., 2008). In terms of in-service teacher training, particularly during online teaching, solutions such as personalized online workshops, characterized by “just-in-time learning,” self-assessment, and flexibility in content and scheduling, should be considered to leverage the benefits of online learning wherein teachers are the students (Rhode et al., 2017). At the intersection of theory and practice, the importance of “quick response research,” such as that conducted in the present study, can assist in understanding the needs and experiences of teachers as they transition from traditional to digital (online or blended) learning modes (Lockee, 2021).

### Limitations and future studies

This study has several limitations. First, the major limitation of this present study is related to the sampling strategy, which relied upon with the assistance of the local government authorities, which may have unintentionally influenced teachers’ responses, as well as their willingness to complete a follow-up survey after 2 months (i.e., the sample for the longitudinal portion of the study). It should be noted that, although the subjects of the longitudinal study and the subjects of the cross-sectional study shared a similar demographic background and reported similar levels of PNT of online teaching, the changes in psychological distress were different for the two samples. Whether or not this situation was due to official assistance provided through participation in the online survey is still uncertain. We suggest that future research should initiate longitudinal monitoring of teachers’ mental health after they return to face-to-face teaching, and explore related factors which can influence teachers’ psychological well-being and intention to continue in specific teaching tasks. Second, given that PNT of online teaching describes a perception toward one’s working environment, exploring the effect of school management as a school-level variable to represent a work/environmental factor related to teachers’ online teaching PNT is another potential area for future research. Third, while the present study focused on the thwarting of teachers’ psychological needs as a risk factor, measures of psychological need satisfaction may be explored by future studies to examine its potential as a protective factor and its relationship with other measures of psychological well-being regarding the use of online teaching during both times of distress, as well as under normal working conditions. Finally, while the present study was concerned with the evaluation of the direct effects of PNT of online teaching in terms of both immediate effects (including psychological distress and satisfaction with online teaching) as well as delayed effects (intention to continue using online teaching), future studies can further evaluate potential mediation and moderation effects. Moreover, future studies may test models that include alternative predictor and outcome variables in relation to the construct of PNT of online teaching.

### Conclusion

In this present study, we systematically evaluated the psychometric properties of the PNTSOT instrument, as developed by Yi et al. (2021) focusing on establishing the longitudinal reliability and validity of the scale. The results demonstrated that the PNTSOT had ideal internal reliability and factorial validity among middle and primary school teachers. Moreover, the test–retest reliability was also acceptable and the tests of longitudinal measurement invariance further confirmed that the PNTSOT can be effectively used to compare the perceptions of PNT among different occasions. As such, we conclude that the PNTSOT can be relied upon as a valid instrument to predict teachers’ PNT before or during the launching of online teaching, or other similar interventions. The results also remind us that, in addition to providing continual training related to online teaching, school administrators must provide more flexibility and autonomy during online teaching. Moreover, based on our findings, that the impact from PNT of online teaching is persistent and long-term, we suggest that school counselors could provide differentiated and personalized assistance to those teachers who express higher levels of psychological need thwarting during the mandatory online teaching or other similar interventions.

As noted in our results on the change in psychological distress between the two occasions, improvement in psychological well-being among schoolteachers was not found in either the cross-sectional or longitudinal data. This finding is consistent with the research from Spain (Ozamiz-Etxebarria et al., 2021a) and Denmark (Nabe-Nielsen et al., 2021). In the former study, high psychological distress still occurred when the school initially reopened, based on cross-sectional data (Ozamiz-Etxebarria et al., 2021a); while in the latter study, adopting longitudinal data, poor mental health outcomes increased extensively (from 27 to 84%) from the time of school closure to school re-opening (Nabe-Nielsen et al., 2021). From these studies, scholars identified teachers’ concerns about infection and increased efforts to prevent contagion as major causes of poor mental health after campus reopening. Research into issues of teacher well-being and related influences must continue, even after vaccines become widely available, since pandemics are unpredictable and, along with other potential crises (e.g., sudden changes in the status quo for teachers) which require the adoption of new technologies or techniques, will certainly impact, and potentially thwart, teachers’ psychological needs.

As we move through the next stages of the pandemic (a post-COVID-19 era), we can take advantage of the lessons learned during the pandemic as an opportunity to better evaluate the potential future of other innovations and evolutions in educational practice that promote online teaching and other interventions which can enhance teaching and learning (Gouëdard et al., 2020; UNESCO, 2020; Munoz-Najar et al., 2021; Rodriguez et al., 2021). School leaders are encouraged to acknowledge that mandatory online teaching is already very stressful for teachers, and that providing sufficient autonomy is key to maintaining teachers’ willingness to continue using online teaching in the future, highlighting the role school leaders can play in such periods of uncertainty and psychological risk.

## Data availability statement

The raw data supporting the conclusions of this article will be made available by the authors, without undue reservation.

## Ethics statement

The studies involving human participants were reviewed and approved by Jianxi Psychological Consultant Association. The patients/participants provided their written informed consent to participate in this study.

## Author contributions

I-HC and C-YL: conceptualization, formal analysis and project administration. I-HC, X-MC, and C-YL: methodology. I-HC and X-MC: validation. I-HC, K-YZ, and Z-HW: investigation. I-HC and X-LL: resources. X-LL: data curation. I-HC and JG: supervision, writing—review and editing and writing—original draft preparation. I-HC: funding acquisition. All authors have read and approved the final manuscript.

## Funding

This research was supported by the Anhui Province Philosophy and Social Science Planning Project “Evidence-Based Decision Making and Practice Research on Comprehensive College Entrance Examination Reform in Anhui Province” (Project No.: AHSKZ2021D12) and the 2022 Shanghai Philosophy and Social Science General Project for Educational Planning “Research on the TPACK Development Mechanisms and Enhancement Paths of College Teachers in the Post-Epidemic Era” (Project No.: A2022014).

## Conflict of interest

The authors declare that the research was conducted in the absence of any commercial or financial relationships that could be construed as a potential conflict of interest.

## Publisher’s note

All claims expressed in this article are solely those of the authors and do not necessarily represent those of their affiliated organizations, or those of the publisher, the editors and the reviewers. Any product that may be evaluated in this article, or claim that may be made by its manufacturer, is not guaranteed or endorsed by the publisher.
