# Supplementary material for: Evaluating the immediate and delayed effects of psychological need thwarting of online teaching on Chinese primary and middle school teachers’ psychological well-being
Source: Front Psychol. 2022 Aug 16;13:943449. doi: 10.3389/fpsyg.2022.943449 (PMC9424862; doi:10.3389/fpsyg.2022.943449)
Supplement: Supplementary file 1 [file Data_Sheet_1.docx]

Supplementary Table 1. Pearson correlations among the observed variables of the three subscales of the PNTSOT

| Yi et al. (12) (*n* = 9,030) | | | |
| --- | --- | --- | --- |
|  | 1. Autonomy  Thwarting | 2. Competence Thwarting | 3. Relatedness Thwarting |
| 1 | 1 |  |  |
| 2 | .57 | 1 |  |
| 3 | .28 | .39 | 1 |
| Time 1 (Cross-sectional data) (*n* = 9,554) | | | |
|  | 1. Autonomy  Thwarting | 2. Competence Thwarting | 3. Relatedness Thwarting |
| 1 | 1 |  |  |
| 2 | .61 | 1 |  |
| 3 | .34 | .43 | 1 |
| Time 2 (Cross-sectional data) (*n* = 4,176) | | | |
|  | 1. Autonomy  Thwarting | 2. Competence Thwarting | 3. Relatedness Thwarting |
| 1 | 1 |  |  |
| 2 | .73 |  |  |
| 3 | .50 | .54 | 1 |
| Time 1 (Longitudinal data) (*n* = 1,642) | | | |
|  | 1. Autonomy  Thwarting | 2. Competence Thwarting | 3. Relatedness Thwarting |
| 1 | 1 |  |  |
| 2 | .62 | 1 |  |
| 3 | .36 | .44 | 1 |
| Time 2 (Longitudinal data) (*n* = 1,642) | | | |
|  | 1. Autonomy  Thwarting | 2. Competence Thwarting | 3. Relatedness Thwarting |
| 1 | 1 |  |  |
| 2 | .72 | 1 |  |
| 3 | .52 | .54 | 1 |

Supplementary Table 2. Comparison between primary and middle school teachers by main observed variables

|  | Group | Mean (SD) | *t*-test (*p*-value) | Effect size: Cohen’s *d* (Interpretation) |
| --- | --- | --- | --- | --- |
| Subscale of autonomy– Measured at Time 1 | Primary school teachers | 3.97 (1.15);  3.88 (1.19) | 2.19 (0.03);  2.20 (0.03) | 0.05 (trivial effect);  0.12 (trivial effect) |
|  | Middle school teachers | 4.02 (1.16);  4.02 (1.20) |  |  |
| Subscale of autonomy– Measured at Time 2 | Primary school teachers | 3.79 (1.38);  3.60 (1.36) | -0.62 (0.54);  0.72 (0.47) | 0.02 (trivial effect);  0.04 (trivial effect) |
|  | Middle school teachers | 3.76 (1.35);  3.65 (1.35) |  |  |
| Subscale of competence– Measured at Time 1 | Primary school teachers | 3.98 (1.26);  3.94 (1.29) | -0.89 (0.37);  -0.22 (0.82) | 0.02 (trivial effect);  0.01 (trivial effect) |
|  | Middle school teachers | 3.96 (1.24);  3.92 (1.27) |  |  |
| Subscale of competence– Measured at Time 2 | Primary school teachers | 3.80 (1.43);  3.70 (1.39) | -0.21 (0.83);  -0.54 (0.59) | 0.01 (trivial effect);  0.03 (trivial effect) |
|  | Middle school teachers | 3.79 (1.40);  3.66 (1.38) |  |  |
| Subscale of relatedness– Measured at Time 1 | Primary school teachers | 2.45 (1.09);  2.38 (1.06) | 6.42 (<0.01);  0.96 (0.34) | 0.14 (trivial effect);  0.05 (trivial effect) |
|  | Middle school teachers | 2.60 (1.11);  2.43 (1.09) |  |  |
| Subscale of relatedness– Measured at Time 2 | Primary school teachers | 2.62 (1.24);  2.51 (1.17) | 0.36 (0.72);  -0.33 (0.74) | 0.01 (trivial effect);  0.02 (trivial effect) |
|  | Middle school teachers | 2.63 (1.22);  2.49 (1.14) |  |  |
| Psychological distress– Measured at Time 1 | Primary school teachers | 20.36 (21.20);  19.56 (19.92) | -1.28 (0.20);  0.21 (0.84) | 0.03 (trivial effect;  0.01 (trivial effect) |
|  | Middle school teachers | 19.79 (20.61);  19.79 (20.95) |  |  |
| Psychological distress– Measured at Time 2 | Primary school teachers | 22.73 (24.59);  19.58 (21.86) | -1.09 (0.27);  -1.30 (0.19) | 0.04 (trivial effect);  0.07 (trivial effect) |
|  | Middle school teachers | 21.86 (24.10);  18.04 (21.57) |  |  |
| Burnout-emotional exhaustion– Measured at Time 2 | Primary school teachers | 3.52 (1.56);  3.51 (1.53) | -0.81 (0.42);  -2.02 (0.04) | 0.03 (trivial effect);  0.11 (trivial effect) |
|  | Middle school teachers | 3.48 (1.52);  3.35 (1.48) |  |  |
| Satisfaction with online teaching– Measured at Time 1 | Primary school teachers | 2.83 (0.68);  2.80 (0.63) | -5.92 (<0.01);  -1.21 (0.23) | 0.13 (trivial effect);  0.07 (trivial effect) |
|  | Middle school teachers | 2.74 (0.69);  2.76 (0.67) |  |  |
| Intention of adopting online teaching in the future– Measured at Time 2 | Primary school teachers | 5.10 (2.69);  4.99 (2.62) | -0.89 (0.37);  1.10 (0.27) | 0.03 (trivial effect);  0.06 (trivial effect) |
|  | Middle school teachers | 5.02 (2.69);  5.15 (2.60) |  |  |

*Note*: the first value in each cell is based on cross-sectional data and the second value is based on longitudinal data.

Supplementary Table 3. Model fit indices and model comparisons for primary and middle school teachers on the PNTSOT

|  | *χ^2^* (*df*) | CFI | RMSEA | SRMR | Δ*χ^2^* (*df*) | ΔCFI | | ΔRMSEA | ΔSRMR |
| --- | --- | --- | --- | --- | --- | --- | --- | --- | --- |
| **Factor structure** |  | | | | | | | | |
| Primary school teachers (11 items) | 117.930 (41) | 0.994 | 0.040 | 0.056 |  |  |  | |  |
| Middle school teachers (11 items) | 225.361 (41) | 0.964 | 0.096 | 0.075 |  |  |  | |  |
| Primary school teachers (12 items) | 365.204 (51) | 0.981 | 0.073 | 0.083 |  |  | |  |  |
| Middle school teachers (12 items) | 399.758 (51) | 0.943 | 0.119 | 0.110 |  |  | |  |  |
| **Measurement invariance**  **(primary and middle school teachers)** |  |  |  |  |  |  | |  |  |
| M1^a^ | 311.46 (82) | 0.988 | 0.058 | 0.075 |  |  | |  |  |
| M2 ^a^ |  |  |  |  | 3.210 (8) | **0.000** | | **-0.003** | **-0.010** |
| M3 ^a^ |  |  |  |  | -45.120 (11) | **0.003** | | **-0.010** | **0.000** |
| M1 ^b^ | 756.79 (102) | 0.972 | 0.088 | 0.110 |  |  | |  |  |
| M2 ^b^ |  |  |  |  | 15.370 (9) | **0.000** | | **-0.003** | **0.000** |
| M3 ^b^ |  |  |  |  | -324.88 (12) | **0.014** | | **-0.023** | **-0.030** |

^a^ 11 item version; ^b^ 12 item version; M1= Configural Model, M2= Loadings Constrained as Equal, M3= Loadings and Thresholds Constrained as Equal; CFI = comparative fit index; RMSEA = root mean square error of approximation; SRMR = standardized root mean square residual; Supported measurement invariance values are in bold, i.e., ΔCFI > −0.01; ΔRMSEA < 0.015; ΔSRMR < 0.03 (for factor loading) or ΔSRMR < 0.01 (for item threshold).
